# Army Schools of Hygiene

**Published:** 1921-02-05

**Authors:** 


### Army Schools of Hygiene.

An Army Order announces that Command Schools of Hygiene are to be established at Aldershot (when the transfer of the school at Blackpool is effected); Hertford Barracks, Hertford; Strensall Camp, near York; Dreghorn Camp, near Edinburgh; Hilsea Hutments, Portsmouth; and Carrickfergus. These schools are to be permanent centres of instruction for staff and regimental officers and for regimental sanitary *personnel*, and they will maintain close liaison with neighbouring units in order to facilitate the demonstration of the principles of practical hygiene on model lines. Four classes for officers will be held annually, and the classes for regimental non-commissioned officers and men will extend over a period of three weeks. Classes for *personnel* of the Royal Air Force will run concurrently with the Army classes, and Territorial Force sanitary companies will be attached to the Command Schools for instruction during their period of training.

